# Myocardial Infarction as an Initial Presentation of Essential Thrombocythemia With Calreticulin (CALR) Mutation (None Type 1, None Type 2)

**DOI:** 10.7759/cureus.33612

**Published:** 2023-01-10

**Authors:** Mohammad Afana, Mohammad Abu-Tineh, Anil Ellahie, Omar Ismail, Deena Sideeg, Afaf Albattah, Mohamed A Yassin

**Affiliations:** 1 Department of Oncology, Hematology and Bone Marrow Transplantation (BMT) Section, National Center for Cancer Care and Research, Doha, QAT; 2 Department of Hematology, National Center for Cancer Care and Research, Doha, QAT; 3 Department of Hematology and Medical Oncology, National Center for Cancer Care and Research, Doha, QAT

**Keywords:** myeloproliferative neoplasm, thrombosis, calreticulin, essential thrombocythemia, myocardial infarction

## Abstract

Essential thrombocythemia (ET) is one of the classical Philadelphia-negative myeloproliferative neoplasms with different mutations that can be associated with it, like Janus kinase 2 (JAK2), myeloproliferative leukemia protein (MPL), and Calreticulin (CALR) (types 1 and 2). However, there is a lack in the literature concerning other types of CALR mutations and their clinical significance and prognosis. Here we report a 42-year-old male with type 2 diabetes who presented with an inferior ST-elevation myocardial infarction and thrombocytosis. The diagnosis of ET with CALR (neither type 1 nor type 2) was confirmed, which suggests the pathognomonic feature of this mutation.

## Introduction

With an annual prevalence of 1.2 to 3.0 per 100,000 people, essential thrombocythemia (ET) is a form of myeloproliferative neoplasm (MPN) characterized by a platelet count (>450 × 10^3/uL) and megakaryocyte proliferation in the bone marrow (BM) [1-3].

The primary clinical manifestations of ET are thrombotic and hemorrhagic complications; Thrombosis can be arterial, which includes strokes, transient ischemic attacks, and coronary artery ischemia, or venous, which includes pulmonary embolisms (PE), or deep vein thrombosis. Bleeding usually occurs when the platelet count is extremely high (>1,500 × 10^3/uL) [4-6].

Thrombosis can involve microcirculation and manifest with tinnitus, dizziness, or migraine. More serious manifestations of thrombosis, like stroke, myocardial infarction (MI), and PE, are well known. This variety of manifestations highlights the morbidity and mortality burden of ET. In acute thrombosis, ET can be missed, especially if the number of platelets is not very high. This has a high morbidity and mortality rate due to the risk of thrombosis coming back. It is important to consider ET in recurrent thrombosis for an unknown reason [1,7,8].

The majority of patients with ET have a mutation in one of three genes: Janus kinase 2 (JAK2) (in 55%), myeloproliferative leukemia protein (MPL) (in 4%), or Calreticulin (CALR) (in 15-24% of patients). Two predominant variants account for 85% of CALR mutations: type 1, characterized by a 52-bp deletion, and type 2, characterized by a 5-bp insertion. However, another variant has been described in the literature, and their pathognomonic features are confirmed by clinical correlation. Patients with the CALR type 1 mutation are more susceptible to thrombosis compared to CALR type 2. JAK2-mutated patients with ET have a major thrombotic event, double what is seen in CALR-mutated patients [1,9,10]. We are reporting a patient who presents with ST-segment elevation myocardial infarction (STEMI) as the initial manifestation of CALR-mutated ET (neither type 1 nor type 2).

## Case presentation

A 42-year-old male presented to the emergency department with acute, left-sided chest pain that was burning in nature and associated with nausea, sweating, and shortness of breath. His medical history revealed that he had type 2 diabetes mellitus for ten years and was poorly controlled (HbA1c 9.1%; normal range 4.8-6.6). The patient is a two-pack-year smoker as well. He had a family history of coronary artery disease in his father; otherwise, he had no personal or family history of dyslipidemia or bleeding disorders.

The vital signs on presentation were as follows: blood pressure 109/78 mmHg, heart rate was 99 beats per minute, and saturation was maintained on room air. The patient was anxious. The cardiac exam was unremarkable. Auscultation of the lungs revealed clear breath sounds. No hepatosplenomegaly was detected on palpation. The patient had a hemoglobin of 16.2 g/dL (13-17 g/dL), a hematocrit of 48.2% (40-50%), a white blood cells (WBC) count of 9x10^3 cells/uL (4x10^3 cells/uL-10x10^3 cells/uL); Absolute neutrophil count of 4.3x10^3 cells/uL (2x10^3 cells/uL-7x10^3 cells/uL), lymphocyte 3.9x10^3 cells/uL (1x10^3 cells/uL-3x10^3 cells/uL), and a platelet count of 1,013x10^3 cells/uL (150x10^3 cells/uL-400x10^3 cells/uL) on the initial complete blood count (CBC).

**Figure 1:**
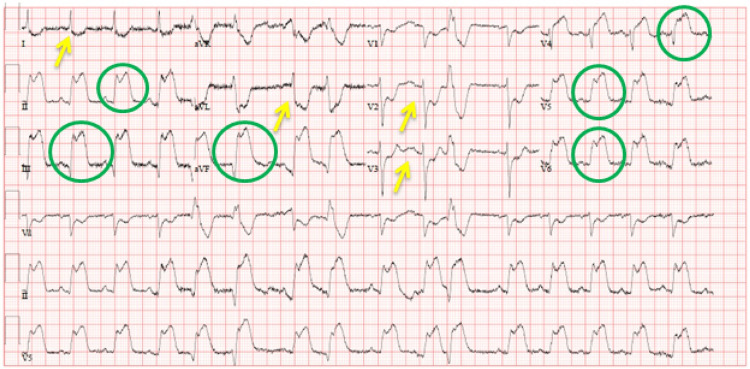
Admission electrocardiogram showing new ST-segment elevation (circle) in leads II, III, aVF, and V4-V6 with ST depression (arrow) in leads I, aVL, and V2-V3, suggestive of inferolateral ST-elevation myocardial infarction

An electrocardiogram showed a sinus rhythm with ST elevation in inferolateral leads (II, III, aVF, and V4-V6) and ST depression in anterolateral leads (I, aVL, and V2-V3) (Figure 1). His initial troponin T level was <10 ng/L (0 ng/L-14 ng/L). A repeat level 6 hours later increased to 2100 ng/L. Triglycerides were 7.1 mmol/L (normal: <1.7 mmol/L), and lactate dehydrogenase (LDH) was 164 IU/L (135-225 IU/L). His electrolytes, urea, and creatinine were within normal limits. The patient was admitted, and thrombolysis with tenecteplase was done without complications as he presented to the peripheral hospital, and it would take more than 2 hours to reach the cath lab in the heart hospital.

Peripheral blood smear showed severe thrombocytosis without an increase in immature myeloid elements. Molecular studies were negative for JAK2 mutations in V617F and positive for a CALR mutation (2 bp insertion mutation within exon 9 of the CALR gene). Testing for Philadelphia chromosome (BCR-ABL) was negative. An abdominal ultrasound was done, and it was unremarkable as well. The patient was scheduled for outpatient coronary angiography. He was discharged on medical treatment, including dual antiplatelet therapy with aspirin and clopidogrel.

The patient was evaluated in the hematology clinic before the coronary angiography and BM procedures were done. It was consistent with ET.

The patient was started on Hydroxyurea with a plan to go for coronary angiography once platelets were below 600 x 10^3/uL. The patient was lost to follow-up, and no coronary angiography was done in our hospital.

## Discussion

Thrombotic complications are the primary cause of death in ET; they can be arterial or venous. The risk for thrombosis is higher in patients older than 60 years, those with prior thrombosis, and those with JAK2/MPL mutations [4,5,9].

Montanaro et al. report a 1.4% annual rate of thrombotic events in a group of 1,144 ET patients, with 25% of all thrombotic events involving the coronary arteries [11].

The underlying pathogenesis is unclear; it may be due to platelet count and its aggregation.

Coronary arteries thrombosis in ET commonly affects the left anterior descending artery as per literature, although involvement of the right coronary artery has been reported [5,6].

The CALR mutation was discovered in 2013; previous literature described the association between thrombosis and the two predominant variants of the CALR mutation (type 1 and type 2); to our knowledge, other CALR mutation variants have yet to be evaluated in relation to thrombosis in ET [12].

In our case, we describe a patient who was found to have a CALR variant (none type 1, none type 2; 2 bp insertion mutation within exon 9) and was presented with an acute STEMI. Our case demonstrates the possibility of thrombosis associated with other CALR mutation variants in ET patients, which remains one of the unmet needs and unanswered questions in MPN, which we attempted to clarify [13-16].

## Conclusions

In conclusion, the pathogenesis of different variants of CALR-mutated ET concomitant with MI is yet to be evaluated.

Our case adds to the growing literature on thrombosis in ET patients in general and specifically to thrombosis in variant CALR mutated ET, suggesting an association of the different variants of CALR with thrombosis. Further reports of similar CALR-mutated ET will support the pathogenesis of such mutation with thrombosis in the future.
